# Neurofilament Light (NF-L) Chain Protein from a Highly Polymerized Structural Component of the Neuronal Cytoskeleton to a Neurodegenerative Disease Biomarker in the Periphery

**DOI:** 10.24966/AND-9608/100056

**Published:** 2021-10-13

**Authors:** Yuhai Zhao, Lisa Arceneaux, Frank Culicchia, Walter J Lukiw

**Affiliations:** 1 LSU Neuroscience Center, Louisiana State University Health Science Center, New Orleans LA 70112, USA; 2 Department of Cell Biology and Anatomy, LSU Health Science Center, New Orleans LA 70112, USA; 3 Department of Neurosurgery, Louisiana State University Health Science Center, New Orleans LA 70112, USA; 4 Department of Ophthalmology, Louisiana State University Health Science Center, New Orleans LA 7011, USA; 5 Department of Neurology, Louisiana State University Health Science Center, New Orleans LA 70112, USA

**Keywords:** Aging, Alzheimer’s disease (AD), AD biomarkers, AD diagnostics, Degradation, Neurofilament light (NF-L) chain protein, Neuronal atrophy, Synapsin-2 (SYN-II)

## Abstract

Neurofilaments (NFs) are critical scaffolding components of the axoskeleton of healthy neurons interacting directly with multiple synaptic-phosphoproteins to support and coordinate neuronal cell shape, cytoarchitecture, synaptogenesis and neurotransmission. While neuronal presynaptic proteins such as synapsin-2 (SYN II) degrade rapidly via the ubiquitin-proteasome pathway, a considerably more stable neurofilament light (NF-L) chain protein turns over much more slowly, and in several neurological diseases is accompanied by a pathological shift from an intracellular neuronal cytoplasmic location into various biofluid compartments. NF-L has been found to be significantly elevated in peripheral biofluids in multiple neurodegenerative disorders, however it is not as widely appreciated that NF-L expression within neurons undergoing inflammatory neurodegeneration exhibit a significant down-regulation in these neuron-specific intermediate-filament components. Down-regulated NF-L in neurons correlates well with the observed axonal and neuronal atrophy, neurite deterioration and synaptic disorganization in tissues affected by Alzheimer’s disease (AD) and other progressive, age-related neurological diseases. This Review paper: **(i)** will briefly assess the remarkably high number of neurological disorders that exhibit NF-L depolymerization, liberation from neuron-specific compartments, mobilization and enrichment into pathological biofluids; **(ii)** will evaluate how NF-L exhibits compartmentalization effects in age-related neurological disorders; **(iii)** will review how the shift of NF-L compartmentalization from within the neuronal cytoskeleton into peripheral biofluids may be a diagnostic biomarker for neuronal-decline in all cause dementia most useful in distinguishing between closely related neurological disorders; and **(iv)** will review emerging evidence that deficits in plasma membrane barrier integrity, pathological transport and/or vesicle-mediated trafficking dysfunction of NF-L may contribute to neuronal decline, with specific reference to AD wherever possible.

## 4. Introduction

Neurofilaments (NFs) are 10 nm diameter, neuron-specific filaments belonging to the type IV intermediate filament (IF) family of structural cytoskeletal polymers in neurons. NFs consist of 3 major classes referred to as the NF triplet: the neurofilament light (NF-L; ~ 60 kDa) chain, the neurofilament medium chain (NF-M; ~90 kDa) and the neurofilament heavy chain (NF-H; ~115 kDa). The stoichiometry of these 3 NF subunits varies slightly, but is approximately 5:3:1 for NF-L:NF-M:NF-H in large neurons of the human brain neocortex; large neocortical layer 3 and 5 pyramidal neurons of the neocortex are specifically targeted and devastated by the Alzheimer’s disease (AD) process [1–4]. Together with the ~ 25 nm diameter microtubules and ~ 7 nm diameter microfilaments and accessory filament proteins such as α-internexin and peripherin, the NF triplet forms a highly dynamic neuronal cytoskeleton contributing to the cytoarchitecture of the neuron [2,3,5–8]. The NF-L chain protein is the most abundant NF in axons, forming the core of the NF bundle, and is a critical scaffolding component of neurite extensions, the primary regulator of the radial diameter of axons and the overall shape of the neuronal cytoskeleton. In doing so NFs support synaptic function and organization by enabling electrical signal transmission along axons and across dendrites and synapses. IF-bound NF-L is therefore an important integral contributor to the structural organization of the ~90+ billion neurons of the brain and spinal cord of the central nervous system (CNS), and also appears to contribute in part to the heterogeneous axonal structures of the sensory and motor neurons of the peripheral nervous system (PNS) [3,4,8–12].

Cytoskeletal disorganization and synaptic degeneration are early and widespread pathogenic events in neurodegenerative disorders with reduced levels of neurofilament and pre- and postsynaptic proteins being recognized as a critical feature of AD pathophysiology. NF-L and synaptic proteins in peripheral biofluids provide valuable diagnostic, prognostic and disease-monitoring biomarkers [8,10,12–20]. While SYN-II and in particular NF-L are found to be abundant in all healthy neurons of the CNS and PNS, diseases that exhibit neuronal degeneration with axonal and synaptic damage display increases in the CSF- and serum-levels of NF-L and other disease-associated proteins. It has been known for at least 25 years that patients with AD, amyotrophic lateral sclerosis (ALS) and multiple sclerosis (MS) slowly release NF-L filament proteins from diseased and atrophied neurons into the CSF [21–23]. AD and ALS patients exhibit increased levels of NF-L protein in their CSF as verified using ELISA, from about ~ 2.5–15-fold over age-matched controls, thus providing a peripheral biofluid indicator of axonal damage and neuronal cell demise. This finding has been suggested to serve as a useful biomarker and indicator for the degree of neurodegeneration as well as a monitor for the progression of both all-cause and AD-type dementia [12,19–22,24]. In tissue biofluids such as the CSF, blood serum and vitreous, well outside of the actual neuronal cell body, the widely observed elevations in NF-L in biofluids strongly suggest a very active neuropathology-related NF-L mobilization, translocation and trafficking beyond the confines of the neuronal plasma membrane and neuronal cytoarchitectural compartments.

## 5. NF-L and SYN-II Expression and Function

In our laboratory the expression of NF-L and SYN-II at both the mRNA and protein level, and the translocation and trafficking of NF-L and SYN-II have been studied together for multiple, highly interactive and sometimes overlapping reasons over the last ~ 35 years. NF-L and SYN-II: (i) are among the most abundant neuron-specific transcripts within healthy neurons of the human association neocortex and are especially abundant in the human hippocampal CA1 and limbic system which are the same anatomical regions targeted by Alzheimer’s disease (AD) neuropathology [8,10,12,25–30]; (ii) both play roles in adult neurogenesis, neuronal development and synaptic physiology that are in-dispensable in nervous system development [3,31,32]; (iii) NF-L and SYN-II expression are found to be hyper-sensitive to AD-type change; as two of the most abundantly expressed neuron-specific phosphoproteins in pyramidal layer 5 neocortical neurons, the largest neurons in the brain (control neocortical layer 5 neurons have normal perikaryal volumes averaging up to 999 μm^3^ in control neocortex); these are very large neurons highly susceptible to AD-induced neuropathology both in human AD and in stressed human neurons (transplantation grade) in primary co-culture [30,33,34]; (iv) are respectively key axonal and presynaptic components involved in the neurotransmission mechanism and the neurotransmitter release cycle [30,35]; (v) NF-L and SYN-II are key supporters of the cytoskeleton synaptic cytoskeletal system in neural development and in both neuronal structure and function and utilize the common transcription factor activators zif268/egr-1/Krox-24 in their promotors (see below); both NF-L and SYN-II may be co-regulated in neuronal gene expression during development and appear to be deregulated together during neurodegeneration [5,30,36,37]; (vi) are approximately the same size and mass (NF-L size 543 amino acids; NF-L molecular mass: 61517 Da; SYN-II size: 582 amino acids; SYN-II molecular mass: 62996 Da) and their mRNAs and protein can be analyzed, quantified and compared with extremely high sensitivity using immunocytochemistry and Western gel analysis, and also by using ELISA, RT-PCR, Northern blots, RNA sequencing and array-based technologies (www.genecards.org/cgi-bin/carddisp.pl?gene=NEFL; www.genecards.org/cgi-bin/carddisp.pl?gene=SYN2#; last accessed 28 September 2021; [30]; (vii) are significantly down regulated in progressive neurodegenerative diseases such as amyotrophic lateral sclerosis (ALS), multiple sclerosis (MS) and AD [11,30,33,38,39]; (viii) are two of the most intensively studied of neuron-specific, neurotransmission related phosphoproteins in health and disease, in brain aging, ALS, MS and AD and a considerable literature exists for both NF-L and SYN-II in neurodevelopment, synaptic maturation and molecular and genetic neurobiology [5,15,24,26,33]; www.uniprot.org/uniprot/p07196#ptm_processing; last accessed 28 September 2021); (ix) are two of the most down-regulated neuron-specific phosphoproteins observed in degenerating neurons in inflammatory neurodegeneration both in vitro and in vivo such as in AD brain and in amyloid over-expressing transgenic murine models for AD (TgAD; [30,33]; (x) as functionally related abundant neuronal components NFs and SYN-II co purify in neuronal extracts; interestingly however the similarly sized NF-L and SYN-II have differential decay schemes and kinetics with NF-L accumulating in the periphery while SYN-II appears to be locally degraded [30,40,41]; (xi) deficits in NF-L, SYN-II and other related neuron-specific elements are not only associated with atrophied or degenerating neurons but are also colocalized within the core and periphery of the senile plaque (SP) and/or neurofibrillary tangles (NFT), two key lesions that characterize AD brain neuropathology [25, 30,42–47]; (xii) disruption in NF-L and SYN-II expression has been associated with over ~200 neurological disorders including ALS, AD, MS, schizophrenia, autism, bipolar disorder, epilepsy, several different types of brain cancer and many other human diseases with a neurological component; (www.malacards.org/search/results/NEFL; www.malacards.org/search/results?query=SYN2; last accessed 28 September 2021); and (xiii) both NF-L and SYN-II expression are under transcriptional control by the CNS-abundant zif268/egr-1/Krox-24 family of transcription factors that are known to be involved at the coordinated transcription level in a variety of higher order neurological processes within the human CNS; these include learning, memory, synaptic network formation and plasticity in the mammalian brain. Again both NF-L and SYN-II expression are observed to be down-regulated in multiple neuropsychiatric disorders involving progressive axonal and neuronal atrophy and neurodegeneration [11,30,36,37,39,48] Largely because of the high stability and long half-life of NF-L compared to SYN-II the major focus of this short review will be on NF-L and its translocation from a highly polymerized structural component of the neuronal cytoskeleton to a neurodegenerative disease biomarker in multiple biofluids of the periphery.

## 6. NF-L Dynamics and Trafficking

Under normal homeostatic conditions and in the presence of physiological calcium all NF proteins form relatively insoluble, tight filamentous bundles [3–5,25] and are in constant dynamic motion with continual assembly and disassembly [2,8,24,49]. All NF proteins are substrates of multiple proteolytic systems; for example when axoplasm is exposed to the extracellular fluid (ECF) elevated calcium concentrations activate calpains, calcium-dependent, non-lysosomal cysteine proteases that readily cleave NFs and promote neurofilament degradation [3,50].

Within the NF bundle, human NF-Ls are extremely stable filamentous structures with half-lives in the order of ~ 21 to ~ 55 days or longer [3,31,45]. As mentioned earlier, low levels of NF-L are constantly released from neuronal cell bodies and axons into the ECF that surrounds neurons and this ECF is contiguous with the CSF. Normally neuronal axon-integral NF-L filaments are depolymerized and released in an age-dependent manner, with low levels of NF-L appearing in circulating biofluids even during the healthy aging of the human CNS [12,51–53]. While the significantly increased appearance of NF-L in the CSF and other biofluids is thought to be an IF-mediated signal for dysfunctional neurons or neurons undergoing age-related demise or atrophy, the dynamics and patho-mechanism of NF triplet bundle depolymerization and NF-L liberation remains incompletely understood. NF-L proteins appear to be ‘secreted’, ‘liberated’ or ‘mobilized’ from neurons and subsequently delivered into the interstitial space and ECF surrounding neurons, and next compartmentalized into the CSF, followed by transport into the lymphatic and systemic circulation and blood serum where NF-L concentrations can be detected and analyzed. In the CSF and blood serum NF-L is typically detected with extreme precision using ELISA or other related fluorescent or chemiluminescent techniques to low picogram per milliliter (pg/ml) sensitivity (see https://www.mybiosource.colm/human-elisakits/neurofilament-light-chain-nfl/9399603; last accessed 28 September 2021; https://www.abbexa.com/humanneuro-filament-light-polypeptide-nefl-clia-kit-2; last accessed 28 September 2021). Interestingly, while neuronal presynaptic proteins such as SYN II degrade rapidly via the ubiquitin-proteasome pathway, a considerably more stable NF-L turns over much more slowly, and in neurological disease is accompanied by a pathological shift from an intracellular neuronal cytoplasmic location across multiple membrane barriers into various biofluid compartments [16,20,54].

The biomechanics of NF-L depolymerization, liberation and transport from the neuron’s axonal interior to the surrounding interstitial space and ECF, and through the CSF into the systemic circulation, including the suspected role of micro-vesicular transport of IF proteins is also not well understood in either its neurobiological or molecular complexity [55–59]. An augmentation or interference of axonal IF trafficking by disorganized or modified NFs, and deficits or alterations in NF transport or translocation across the neuronal plasma or cytoplasmic membranes has been proposed as one possible mechanism of NF-mediated neuropathology in both AD and ALS and perhaps other neurological disorders [5,39,60–65]. The increasing abundance of NF-L in biofluids including the ECF, CSF, blood serum and/or vitreous fluid of the eye may serve as a diagnostic or prognostic biomarker for evaluating disease progression in different nervous system disorders for neurodegenerative diseases and/or in transgenic animal models of progressive age-related neurodegeneration [13,30,39,57–59,66].The most recent research suggests that NF-L presence in peripheral biofluids may be a stronger biomarker for all cause generalized decline in neuronal structure and integrity, defective network signaling and progressive neurodegeneration and/or neurodegenerative dementia [4,18,24,29,67,68].

## 7. NF-L as a Biomarker and Indicator of Neuropathology

NF-L presence and abundance in the CSF and/or peripheral blood serum may not only be useful diagnostically for detecting the earliest signs of neurodegeneration but also: (i) in evaluating the degree and severity of progressive neurodegenerative damage and pathological change in the aging nervous system; (ii) may be informative as an indicator of neuronal and/or axonal atrophy and related morphologic changes in the brain at both the microscopic and macroscopic level; and (iii) may be further useful as a general biomarker of neurological disease progression in multiple nervous system disorders, including the evaluation of treatment efficacy for traumatic brain injury (TBI), ischemic stroke and inflammatory neurodegeneration [4,47,69,70]. Importantly, deficits in NF-L abundance within the neuronal cytoskeleton and cytoarchitecture may not only have structural and functional implications for neuronal integrity and function but is also linked to excessive extra-neuronal NF-L presence in multiple neurodegenerative diseases exhibiting both progressive neurite degeneration and demyelination. The list of neurological disorders where NF-L has been found to be enriched in biofluids such as the CSF is both significant and remarkable. Such disorders include AD [16,24,71,72], amyotrophic lateral sclerosis (ALS; [24]); generalized brain atrophy [71]; frontotemporal dementia (FTD) [14,68]; idiopathic normal pressure hydrocephalus (iNPH); multiple sclerosis (MS) [13]; lysosomal storage diseases such as Gaucher’s disease (GD); spinal muscular atrophy (SMA); sub-cortical arteriosclerotic encephalopathy (SAE) [68,73]; hereditary sensory-motor neuropathy (HSMN); Parkinson’s disease (PD); polyglutamine in CAG trinucleotide repeat disorders; both ischemic and hemorrhagic stroke; vascular dementia (VaD) and other relatively rare neurodegenerative disorders such as neuronal ceroid lipofuscinosis type 2 (CLN2 disease) and rare prion diseases that include Creutzfeld-Jacob disease (CJD) [4,10,12,14,24,40,53,68,74,75]. In fact defective NF-L and SYN-II signaling have been linked to over 200 human diseases and because each of these disease states involve these 2 neuron-specific functionally-linked cytoskeletal and synaptic phosphoproteins and the majority of these disorders have a neurological component (www.malacards.org/search/results/NEFL; www.malacards.org/search/results?query=SYN2; last accessed 28 September 2021).

Pathologically, NF-L filaments are further found within and/or associated with abnormal assemblies and disease-related accumulations and senile plaque lesions associated with amyloid beta (Aβ) peptide deposits in AD and synuclein in PD; NF-L proteins have also been found to directly associate with superoxide dismutase 1 (SOD1), TAR DNA-binding protein 43 (TDP43), neuronal RNA-binding FUS proteins, optineurin (OPTN), ubiquilin 2 (UBQLN2), dipeptide repeat protein (DRP), and synapsins and other pre-synaptic phosphoproteins [4,5,10,12,14,27,30,33,40,53,56,76]. Whether NF-L is covalently locked within these pathological lesions or can dissociate away and contribute to biofluid NF-L pools is not known. When axonal and dendritic damage, inflammatory, neurodegenerative, traumatic or vascular injury occurs in multiple neurological disorders such as AD, ALS, PrD, VaD there often appear to be increases in NF-L accumulation in the CSF which are directly proportional to the severity of these progressive age-related neurodegenerative disorders [4,12,24,38,49,56,77]. Interestingly in MS, CSF NF-L concentrations have been shown to correlate with clinical and radiological outcomes, making NF-L presence potentially useful for monitoring patient response to different MS therapies [11,22,49,51,57].

## 8. Compartmentalization of NF-L in Neurodegenerative Disease

While highly polymerized NF-L filaments are the major component of type IV IF scaffolding proteins in neurons and the most abundant neuron-specific filament class at the core of the NF triplet, their disease-related occurrence in circulating biofluids outside of the CNS may seem paradoxical. Normally large ~ 61.5 kDa proteins like the NF-L protein filaments exist as a highly organized polymer stably bundled and confined within the axonal cytoskelton of neurons, and have difficulty in passing through intact plasma membranes. However, damaged plasma membranes and leaky biophysiological barriers of the extensive neurovascular system of the human CNS appear to allow filamentous protein passage across biological membranes under both pathological conditions and with aging [62,65,78]. There is a strong correlation of brain endothelial neurovascular cell damage and leakiness of biological membrane structure associated with increased plasma membrane porosity and increased passage of normally excluded biomolecules, neurotoxins and multiple pro-inflammatory mediators such as lipopolysaccharide (LPS) and NF-L [49,62,65,78–82]. Recent reports indicate that higher serum NF-L levels are also associated with greater impairment of blood-brain barrier (BBB) integrity as measured by albumin quotient assay (at present one of the most reliable biomarkers for estimating the BBB permeability) coupled with brain MRI studies [49,83]. Moreover, as an index of neuronal degeneration and functional decline, higher concentrations of NF-L in both the CSF and plasma are associated with reduced [^18^F]FDG uptake and hypometabolism in AD brain and contribute to the diagnosis of neuronal atrophy and neurodegeneration in serum amyloid-positive individuals with AD [84]. There is evidence for a mechanistic link between inflammation and BBB integrity and function in both neurodevelopmental and neurodegenerative disease [85,86]. Another recent and related report provides evidence that in the vitreous fluid of the eye NF-L is positively associated with increased levels of Aβ42 peptide, inflammatory cytokines such as interleukin-15 (IL-15), monocyte chemoattractant protein-1 (MCP1), and vascular proteins such as vascular endothelial growth factor receptor-1 (VEGFR1), vascular cell adhesion molecule-1 (VCAM-1) and intracellular adhesion molecular-1 (ICAM-1) suggesting the clinical utility of obtaining vitreous samples as a possible source of protein array-based diagnostic testing for AD or other age-related, AD-like neurological disorders [86].

## 9. NF-L Depolymerization and Degradation in Neurological Disease

**Figure 1:**
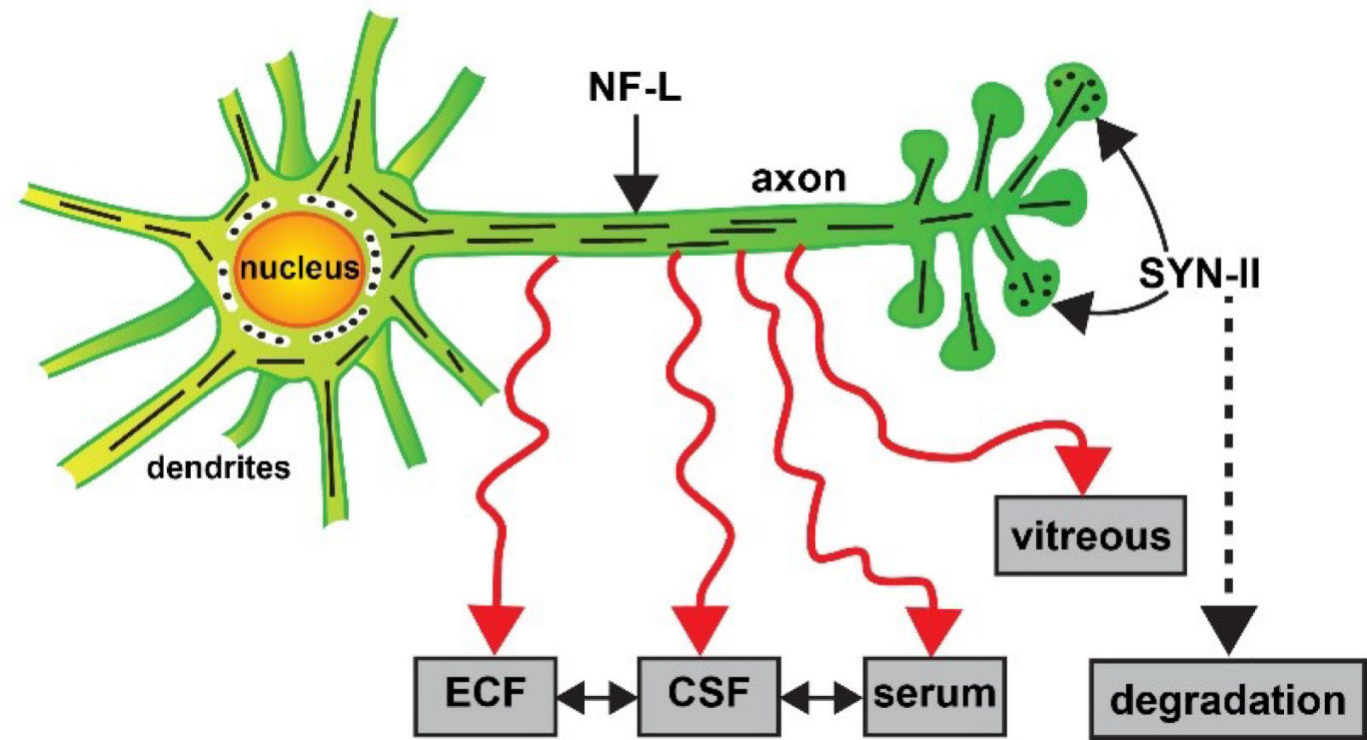
Alternate fate of the neuron-specific neurofilament light (NF-L) chain and synapsin 2 (SYN-II) proteins in degenerating neurons. Highly schematicized depiction of NF-L protein as an important intermediate filament (IF) component of the axon and cytoarchitecture that supports dendrites and synaptic structures of neurons and a key player in cytoskeletal dynamics; a critical pre-synaptic phosphoprotein SYN-II is involved in tethering synaptic vesicles to one another, in the regulation of synaptic vesicle release and trans-synaptic signaling neurons, in the linkage and association of synaptic vesicles to the cytoskeleton; in doing so SYN-II modulates neurotransmitter release across the presynaptic membrane in both the central and peripheral nervous system (CNS, PNS); NF-L is also an integral component and critical organizer of synapsin-enriched synapses that modulate neurotransmission, behavior and memory; NF-L and SYN synaptic proteins together serve highly interactive roles; both are significantly depleted in dystrophic and atrophied neurons in AD and in other related neurodegenerative disorders [3,30,31,40,76]. SYN-II proteins (dark black dots at the pre-synapse) are very labile (half-life ~ 24 hr), exhibit high rates of turnover, decomposition and decay, and are rapidly degraded via the ubiquitin-proteasome pathway [54]. On the other hand neuronal NF-L filament proteins (short black lines) are extremely stable filamentous proteins (half-life ~ 504 to ~ 1320 hr or longer; [3,45], normally insoluble, resist degradation, and can leak out of the plasma membrane of degenerating neurons to subsequently appear in peripheral biofluids in the ECF, CSF, blood serum and/or vitreous of the eye (red wiggly eyes); the mobility of NF-L between ECF, CSF and serum compartments is not well understood; elevated NF-L abundance in peripheral biofluids has been observed in AD, ALS, CJD, CLN2, FTD, GD, iNPH, MS, neuronal atrophy, VaD and other forms of progressive neurodegenerative disease (see manuscript text; [11,14,17,25,47,68,70,86,94]. The abundance of NF-L in biofluids appears to be an indicator of the degree of all-cause neurodegeneration, dendritic and axonal damage, overall neuronal atrophy and neuronal decline; the most recent evidence suggests that this may have diagnostic value to differentiate between AD and FTD or be useful in AD diagnosis and/or prognosis in combination with other biofluid biomarkers such as tau and Aβ peptides [12,19,29,47,67,91,93].

The NF-L chain IF protein monomer is the smallest of the NF triplet yet one of the most abundant and stable neuron-specific components critically essential for maintaining the cytoskeleton of healthy neurons, and thereby the organization of synaptic networks [5,11,13,45,57,76]. It follows that neurodegenerative disorders involving progressive neuronal atrophy and synaptic damage will involve alterations in NF-L and SYN-II neurobiology and abundant evidence continues to support this concept. NF-L and SYN-II proteins have a particularly high density in large pyramidal neurons and myelinated axons extending into the PNS, such as those found in motor neurons [5,10,87]. While SYN-II is rapidly degraded, NF-L release from a fully polymerized filamentous state significantly increases free NF-L concentration in the ECF and CSF in response to CNS axonal damage due to inflammatory, neurodegenerative, traumatic or neurovascular injury (Figure 1). Several studies provide evidence that NF-L concentrations in the CSF may be the highest in brain disorders with subcortical pathology, such as VaD, iNPH and white matter disease [87,88]. As aforementioned, significant free NF-L monomeric concentrations in the CSF have been reported in individuals with FTD, MS and CJD, and more closely correlate with rapidly progressing axonal deterioration and degeneration than in individuals with early stage AD [68,71,87]. It has been suggested that the serial detection of monomeric NF-L over time may be diagnostically useful to differentiate between multiple types of age-related neurological disorders with a similar or indistinguishable clinical presentation [29,67,68,71,87,88]. The most recent extensive correlative analysis on large numbers of neurodegenerative disease patients however indicates that NF-L does not appear to be a highly selective biomarker for any single neurodegenerative diseases or for AD at any stage, but rather an indicator for dendritic damage, axonal deterioration, overall neural cell demise, neurite regression and synaptic atrophy, especially in progressive pathological disorders where large neurons and myelinated axons are involved [47,68,87].

## 10. Concluding Remarks

Age-related neurodegenerative disease continues to present one of contemporary medicine’s most intractable problems in its characterization, diagnosis, prognosis, disease monitoring and clinical management. There are a considerable number of dysfunctional biochemical, neurochemical, cytoskeletal and molecular genetic deficits across the entire spectrum of progressive, age-related degenerative brain diseases such as AD. In contrast to the rapidly degraded synaptic protein SYN-II, alterations in the compartmentalization of significantly more stable NF-L from fixed positions within neurons to their progressive appearance in multiple biofluids lends weight to the understudied idea that there may be transport, translocation and/or trafficking complications of neurofilaments and other essential neuron-specific and/or neuron- or synaptic-associated components which appear to contribute to the progressive and age-related nature of neuronal degeneration. The appearance of the neuron-specific NF-L filament protein in biofluid compartments outside of the neuron is: **(i)** an overall signal for the inception of the functional decline of large CNS neurons; **(ii)** the quantifiable abundance of NF-L in multiple biofluids appears to be an indicator of the degree of this neurodegeneration in both the CNS and/or PNS; and **(iii)** while absolute NF-L abundances are diagnostically useful NF-L presence alone does not appear to be a selective biomarker for any specific neurodegenerative disease. Alternately the appearance of NF-L in biofluids may simply be a consequence of a poorly understood natural decay mechanism for this abundant filament protein in progressive age-related neurological disorders in part due to NF-L’s notable stability and remarkably long half-life (Figure 1) [31,45,57].

Dysfunctional plasma membranes and altered biophysiological barriers including the neuronal plasma membrane-ECF interface and the BBB-systemic circulation endothelial cell barriers are strongly implicated in dysregulated biomolecular trafficking [65,78]. Biophysical barrier disruption may allow depolymerized NF-L mobilization from the neuronal axons and cytoplasm across biophysical interfaces into the ECF, CSF, throughout the systemic circulation, within the vitreous fluid of the eye and perhaps into other biofluid compartments. Multiple laboratories have reported disease-associated deficits in these biological membrane barriers, the BBB, gastrointestinal (GI)-tract intestinal lumen circulatory system barriers and other biophysical interfaces, and the channel and vesicle-mediated traffic passing across them [56,60,62,63,65,78,89,90]. Biofluids including ECF, CSF, blood serum and vitreous fluids contain microRNAs (miRNAs) both free and vesicle-bound that are known to regulate the expression and abundance of neuron-specific components including NF-L [56]. Further quantitative investigation of this vesicular traffic, vesicle content, specific globular and filamentous proteins, lipids, carbohydrates, small non-coding RNA (sncRNA) and other nucleic acids (both RNA and DNA) populating the systemic circulation (including the lymphatic, glymphatic and blood serum), CSF and other biofluids such as saliva and urine are important future goals of AD and neurodegenerative disease research.

Lastly, the detection and quantitation of NF-L in biofluids has become a widely researched, recognized and diagnostically valuable biomarker for the earliest detection and onset of all-cause neural decline in progressive, age-related neurodegeneration, stroke and traumatic brain injury [20,47,91]. It is becoming increasingly clear that NF-L presence in biofluids alone however, is neither specific or selective for AD or for any other progressive age-related neurological disorder [12,29,65,92].The highest value of measuring the abundance of NF-L as a biomarker in CSF may be to differentiate between complex neurological disorders with close and often overlapping clinical presentation, such as assisting the differentiation between FTD (the second most frequent cause of dementia, after AD in patients under the age of 65) from AD, and PD from atypical Parkinsonian syndromes [47,91,93]. Further studies on large age- and gender-matched genetically defined populations, including the application of a more personalized medicine approach for AD diagnosis and treatment [29,75]: (i) should provide an improved understanding of the disease mechanisms involved with NF-L liberation from neurons and NF-L accumulation in biofluids, brain dysfunction and neurodegeneration; and (ii) allow for the discovery of new biomarkers, or defined combinations of biomarkers, for disease prognosis and diagnosis that should engender equally novel therapeutic strategies [94–97].

## 11. Significance of the Research

An overwhelming abundance of research papers report the elevation of neurofilament light (NF-L) chain protein in various biofluids (CSF, blood serum, vitreous fluid and others) in Alzheimer’s disease (AD) and associated neurodegenerative diseases, and their potential role in providing valuable diagnostic, prognostic and disease-monitoring biomarkers. However, not one of these papers discusses the significant down-regulation and altered trafficking of NF-L in atrophied and dysfunctional neurons and in degenerating axons in AD and related degenerative disorders of the brain and CNS. This is Review paper is the first to address the hitherto unappreciated fate of NF-L from a highly polymerized neuron-specific structural component of the neuronal axoskeleton to a monomeric neurodegenerative disease biomarker in the periphery.

## 2. Abbreviations

Aβ: Amyloid beta (peptide)
AD: Alzheimer’s Disease
CSF: Cerebrospinal Fluid
ECF: Extracellular Fluid
ELISA: Enzyme-linked Immunoassay
FTD: Frontotemporal Dementia
GI: Gastrointestinal
IF: Intermediate Filament
NF: Neurofilament
NF-L: Neurofilament Light Chain Protein
PD: Parkinson’s Disease
PNS: Peripheral Nervous System
SYN II: Synapsin 2
VaD: Vascular Dementia
